# Activity in Neurons of a Putative Protocerebral Circuit Representing Information about a 10 Component Plant Odor Blend in *Heliothis virescens*

**DOI:** 10.3389/fnsys.2012.00064

**Published:** 2012-09-27

**Authors:** Bjarte Bye Løfaldli, Pål Kvello, Nicholas Kirkerud, Hanna Mustaparta

**Affiliations:** ^1^Neuroscience Unit, Department of Biology, Norwegian University of Science and TechnologyTrondheim, Norway

**Keywords:** insect olfaction, protocerebral neurons, antenno-protocerebral tracts, lateral protocerebrum, superior protocerebrum, descending neuron and odor mixture

## Abstract

The olfactory pathway in the insect brain is anatomically well described from the antennal lobe (AL) to the mushroom bodies and the lateral protocerebrum (LP) in several species. Less is known about the further connections of the olfactory network in protocerebrum and how information about relevant plant odorants and mixtures are represented in this network, resulting in output information mediated by descending neurons. In the present study we have recorded intracellularly followed by dye injections from neurons in the LP and superior protocerebrum (SP) of the moth, *Heliothis virescens*. As relevant stimuli, we have used selected primary plant odorants and mixtures of them. The results provide the morphology and physiological responses of neurons involved in a putative circuit connecting the mushroom body lobes, the SP, and the LP, as well as input to SP and LP by one multiglomerular AL neuron and output from the LP by one descending neuron. All neurons responded to a particular mixture of ten primary plant odorants, some of them also to single odorants of the mixture. Altogether, the physiological data indicate integration in protocerebral neurons of information from several of the receptor neuron types functionally described in this species.

## Introduction

The intimate relationship between insects and plants relies to a large extent on plant produced chemical cues and sophisticated olfactory and gustatory systems evolved in insects. The importance of these senses is reflected in the numerous sensory organs, sensilla, on the various appendages and the large areas of the brain devoted to chemosensory coding and learning. Important questions in studying olfactory and gustatory mechanisms concern stimulation with relevant odorants and tastants involved in attraction to and selection of host plants, how receptor neurons detect the large diversity of odor and taste molecules, and how innate and learned odor and taste information is handled in the brain, resulting in particular motor output and behavior.

The olfactory system of many insect species is anatomically and partly functionally described with common structures and organization, from receptor neurons projecting in the primary olfactory center, the antennal lobe (AL), to the second order olfactory areas in protocerebrum, like the mushroom bodies (MBs), the lateral- and the superior protocerebrum (LP and SP), and the lateral horn (LH; Galizia and Rössler, 2010). As shown in the fruit fly (*Drosophila melanogaster*) and other species, the olfactory receptor neurons (ORNs) consist of subtypes, each subtype expressing one and the same type of receptor proteins and sending their primary axons to one specific glomerulus, exceptionally two, in the AL (Vosshall et al., 1999; Vosshall and Stocker, 2007). This implies that each glomerulus is considered to receive odor information from one type of ORNs. The number of glomeruli is species specific, like 43 in the fruit fly, 60–67 in moth species, including *Heliothis virescens* (66 in females), and 160 in the honeybee (*Apis mellifera*; Flanagan and Mercer, 1989; Stocker et al., 1990; Berg et al., 2002; Løfaldli et al., 2010). The information is processed in the neural network formed by the synapses between the ORNs and the local and projection neurons (LNs and PNs) in the AL, and modulated by centrifugal neurons also innervating the glomeruli. Like in most insect species studied, the majority of PNs in moths are uniglomerular, innervating single glomeruli, in contrast to the multiglomerular PNs present in a smaller number (Homberg et al., 1988; Stocker et al., 1990; Jefferis et al., 2007; Rø et al., 2007). The processed information is further mediated to the protocerebral areas, mainly via three parallel antenno-protocerebral tracts (APTs), the medial (also called the inner antenno-cerebral tract, ACT), the lateral (also called the outer ACT), and the medio-lateral APT. Whereas the medial and the lateral APT both project in the calyces of the MB and in the LP, but in opposite order, the medio-lateral APT projects in the LP, and the SP, avoiding the calyces of the MB (Homberg et al., 1988; Kirschner et al., 2006; Rø et al., 2007; Galizia and Rössler, 2010).

The MB has been studied particularly in social insects, like honeybees and ants, because of its importance in learning and memory (Heisenberg et al., 1985; Menzel, 2001; Heisenberg, 2003; Gerber et al., 2004). The dendrites of the numerous kenyon cells, receiving information from the PNs of the AL, form the calyces, and the axons the peduncle and the lobe system that is divided into several subsystems of the medial and vertical lobes (Ito et al., 1998; Strausfeld, 2002; Fukushima and Kanzaki, 2009). Several types of extrinsic MB neurons have been morphologically described with dendritic innervations along the pedunculus and the lobes and axon projecting in other protocerebral areas, like the LP and the LH (Homberg, 1984; Ito et al., 1998; Li and Strausfeld, 1999; Tanaka et al., 2008). One physiologically characterized MB extrinsic neuron, the PE1 of the honeybee projecting in LP as well as other areas, is particularly interesting by showing changed responses after odor conditioning (Mauelshagen, 1993; Rybak and Menzel, 1998; Okada et al., 2007). The LP/LH, receiving direct information from the AL as well as via the MB, is considered as a premotoric area, from where descending neurons mediate the information out of the brain. Whereas descending neurons responding to pheromones have been described in the lateral accessory lobes (Kanzaki et al., 1991, 1994), the knowledge is scarce about descending LP neurons responding to plant odors. In flies a ventral area of the LP is shown to house descending neurons and to receive information of different modalities, including olfactory information from the LH (Strausfeld, 1976; Tanaka et al., 2004).

In general, little knowledge exists on the anatomical and functional organization of the LP/LH. Studies in the fruit fly and in *H. virescens* have indicated a stratified projection pattern among m-APT PNs in the LH (fruit fly) and the LP (*H. virescens*); similar and close projections of neurons innervating the same glomerulus and partly overlapping projections of neurons innervating different glomeruli (Marin et al., 2002; Wong et al., 2002; Jefferis et al., 2007; Løfaldli et al., 2010). In *H. virescens* the olfactory projection area in the LP is termed olfactory axis (OA). Regarding the functionality of the LP/LH it has been proposed that the AL-LH(LP) pathway represents a naïve or inexperienced odor processing route from the AL to motor control, compared to the associative and experience dependent MB pathway (Heimbeck et al., 2001; Keene and Waddell, 2007). In a recent study of the fruit fly, Ruta et al. (2010) mapped a pheromone information pathway from the responding PNs in the AL to descending neurons in the LP. They showed that information about the pheromone was transferred to third order LH neurons having dendritic overlap with the PN projections and axonal overlap with the dendrites of descending neurons that responded to stimulation with the pheromone, thus providing a functional pathway from the input to the output of the brain.

Coding mechanisms have particularly been studied in the AL of many insect species, reporting both spatial and temporal principles for odor quality coding (Laurent et al., 1996; Joerges et al., 1997; Galizia and Menzel, 2000; Stopfer et al., 2003; Wang et al., 2003a; Lei et al., 2004; Riffell et al., 2009b). In moths, spatial coding principles are demonstrated by the functional organization of the well defined macroglomerular complex innervated by PNs, some of which show specific responses to single compounds and others integration of the pheromone information (Christensen et al., 1991, 1995; Anton and Hansson, 1994; Berg et al., 1998; Vickers et al., 1998; Christensen and Hildebrand, 2002). Likewise, in the more complex plant odor system of moths, PNs responding specifically to single odorants and others to several odorants have been shown by electrophysiological recordings as well as calcium imaging studies (Müller et al., 2002; Sadek et al., 2002; Skiri et al., 2004; Reisenman et al., 2005; Krofczik et al., 2009; Yamagata et al., 2009; Deisig et al., 2010; Kuebler et al., 2011). Based on calcium imaging, a combinatorial coding mechanism where single odorants and blends elicit specific activity patterns among some glomeruli and neurons in the AL network has also been proposed in the honeybee (Galizia and Menzel, 2000; Galizia and Szyszka, 2008). In contrast, in the locust AL, exclusively containing multiglomerular PNs, odor coding is shown to relay more on the temporal synchronous activities than the spatial activity (Laurent et al., 1996; Perez-Orive et al., 2002). In a recent multiunit recording study in the hawk moth (*Manduca sexta*) stimulation with complex mixtures containing particular odorants derived from host plants elicited synchronous intensity invariant firing patterns among the recorded neuronal units (Riffell et al., 2009a,b). It was found that the mixture was differently represented from the single constituents in a spatio-temporal activity pattern, similar to findings in the honeybee by imaging and electrophysiological recordings from PNs (Deisig et al., 2006; Yamagata et al., 2009). Furthermore, differentiation between information mediated by single odorants and blends have been proposed for the m- and the l-APT, the PNs of the l-APT processing synthetically and the m-APT PNs elementally information about mixtures (Krofczik et al., 2009). In spite of results provided by numerous studies in insects, we need more specific knowledge about the processing of relevant odor information both in the AL and in the protocerebral networks.

The present study focuses on how information about relevant plant odors is represented in neurons projecting in two APT target areas in the protocerebrum of *H. virescens*. It is based on knowledge about primary plant odorants, previously identified by the use of gas chromatography linked to electrophysiological recordings from single receptor neurons (Røstelien et al., 2005). From these long lasting recordings of testing large numbers of naturally produced plant volatile mixtures (“head-space”), we know that ORNs responding to plant odorants in this species belong to distinct functional types, each type responding best to one primary odorant and weakly to a few others of molecular similarity. Furthermore, with one exception, the molecular receptive ranges of the functionally described ORN types do not overlap. This indicates that responses of a central neuron to a primary odorant originate from one particular ORN type. In the present study we have stimulated with selected primary odorants and defined mixtures of them during intracellular recordings from protocerebral neurons, followed by dye injection for morphological characterization. We have asked how information about relevant plant odorants and mixtures are represented among neurons in higher order protocerebral areas. The results revealed that information about a defined 10 component plant odor blend is represented by activity in neurons forming a putative circuit between the MB, SP, and LP in the *H. virescens* brain.

## Materials and Methods

### Insects, stimulation protocol, recordings, and staining

*Heliothis virescens* (Heliothinae; Lepidoptera; Noctuidae) pupae were imported from a laboratory culture (Syngenta, Basel, Switzerland), separated according to sex, enclosed, and kept with access to 0.1 M sucrose solution in an incubator (Refritherm 6E, Struers) on a phase-shifted LD photoperiod (14:10 h) at 25°C. Experiments were performed on 3–6 days old female moths.

Moths were mounted in plastic tubes and immobilized with dental wax (Kerr Corporation, Romulus, MI, USA). Part of head cuticle was removed to expose the superior and lateral parts of the protocerebrum. Large trachea, intracranial-, and antennal muscles were removed to eliminate movements. Glass microelectrodes were pulled with a Flaming–Brown horizontal puller (P97; Sutter Instruments, Novarto, CA, USA), the tips were filled with dye (Micro-Ruby, Invitrogen; 4%) and backfilled with potassium acetate solution (0.2 M). The microelectrodes had a resistance of 150–400 MΩ. Neurolemma was perforated with a tungsten hook to facilitate insertion of the microelectrode prior to super fusion with saline solution.

Neuronal activity was recorded with an Axoprobe amplifier (Molecular devices) and a CED data acquisition unit (Cambridge electronic design) during stimuli protocol application. Olfactory stimuli were applied through glass cartridges as air puffs (0.8 ml/300 ms) into a continuous air stream. Each cartridge contained one of the following primary odorants: 3Z-hexanol, 3Z-hexen-1-ol, 3Z-hexenyl acetate, ocimene, racemic-linalool, geraniol, (+)3-carene, E-verbenol, methyl benzoate, 2-phenyl ethanol, (−)-germacrene D, farnesene, defined blends (PB) with equal amount of each odorant (from a binary to a 12 component mixture), and other blends (ylang oil and magnet), applied (1 μg) to a filter paper (1.5 cm). The primary odorants were selected because of their marked best effect on particular types of ORNs, identified by gas chromatography linked to electrophysiological recordings from single receptor neurons, followed by linked gas chromatography-mass spectrometry (Røstelien et al., 2005). All neurons were tested with purified air as control, and some of them for tactile and taste stimulation (sucrose, quinine hydrochloride, salt, and distilled water) as described in Kvello et al. (2009), as well as sound and light stimulation.

Neurons were iontophoretically stained by passing a 1- to 3-nA depolarizing current of 2 Hz with 0.2 s duration. Complete labeling of the neurons required dye (4% micro-ruby, Invitrogen) injection for 10–15 min. After current injection, the dye was allowed to diffuse over night at 4°C. The brains were dissected in saline solution, fixed in paraformaldehyde (4%) in PBS, and left over night (4°C). Stained neurons were intensified by Streptavidin-Cy3 (Jackson immunoresearch, West Grove, PA, USA; diluted 1:200 in PBS) over night (4°C) before PBS rinsing, dehydration [ethanol series: 50, 70, 90, 96, and 100% (10 min each)] and mounting in methyl salicylate. Most preparations with successful neuronal staining underwent a subsequent background staining with a SYNORF1 protocol; rehydration in ethanol (100, 96, 90, 70, 50%, 10 min each), washing (PBS, 10 min), and preincubation in normal goat serum (NGS; Sigma, St. Louis, MO, USA; 10%) in PBS at room temperature (30 min). Subsequently, the preparations were incubated in a monoclonal antibody against the synaptic protein synapsin (SYNORF 1, Prof. E. Buchner, Würzburg, Germany), diluted in PBS (1:10), and NGS (10%) for 48 h (4°C). Then they were rinsed in PBS (5 × 20 min) before incubation for 24 h (4°C) with a Cy5-conjugated goat anti-mouse secondary antibody (Jackson Immunoresearch; dilution 1:500 in PBS), rinsed again (PBS, 5 × 20 min), and dehydrated before mounted in methyl salicylate on double-sided aluminum slides.

### Visualization, reconstruction, and registration of neurons into the standard brain atlas

Stained preparations were visualized in a laser scanning confocal microscope (LSM 510 META Zeiss, Jena, Germany and a Leica TCS SP5, Leica microsystems). Intracellular fillings were examined with a Plan-Neofluar 20×/0.5 NA dry lens objective and a C-Achroplan 40×/0.8 NA water objective in the Zeiss microscope and a 10×/0.4 NA dry lens objective in the Leica microscope. The intracellular dye was excited by a 543-nm Helium Neon laser and filtered through a bandpass filter BP 565–615 IR (561 HeNe laser in Leica). The fluorescent dye Cy5 was excited by a 633-nm line of argon laser. Preparations were scanned with a resolution of 1024 × 1024 pixels in the *xy*-plane and an interslice distance of 2 μm. Neurons was scanned in several tiles and manually merged in Amira (Visage Imaging). To compensate for the refraction indexes the *z*-axis dimension was multiplied by a factor of 1.6. Gray value image stacks of stained neurons and innervated brain structures acquired from the confocal microscope were examined and semi-automatically reconstructed (Schmitt et al., 2004; Evers et al., 2005; Kvello et al., 2009). The registration of reconstructed neurons into the standard brain atlas (SBA) followed the same procedure as described by Brandt et al. (2005), Kvello et al. (2009), and Løfaldli et al. (2010). Selected brain structures in the preparations with stained neurons were reconstructed as label images and affine and elastically registered to the corresponding label imaged in the SBA. The transformation parameters were then applied to the corresponding reconstructed neuron. The results were then carefully evaluated by comparing the gray value images with the obtained model. The SBA contains only label images of neuropile areas and not cell clusters.

### Physiological analysis

All obtained intracellular voltage traces were processed with a wave form analysis mode in the computer program Spike 2 (Cambridge electronic design). The recordings were reviewed for odor evoked responses and sorted in a response table. Recordings that qualified for further response analyses had to fulfill the following criteria: In addition to control and PB10, the neurons had to be tested for one or more of the four most frequently tested primary odorants, the responses had to be repeated and the recording had to be stable and reliable with low levels of noise. The responses were quantified in bins of 50 ms across all stimuli for all neurons except those with exceptionally wide response windows. The response window was defined as the period between stimulus onset and the first bin where the consecutive 400 ms did not deviate significantly from spontaneous activity. The response window width (number of bins) of a neuron was kept constant across all stimuli, the duration of the longest response determining the width of the response window. Mean frequency and variance of the spontaneous activity were estimated for each neuron. The estimation was based on the weighted average of spike rates from two periods: 200 ms prior to stimuli onset and 1500 ms recorded 400 ms after the end of the defined response windows. The interval of 400 ms after the response window was excluded from the estimation to ensure that neurons had returned to spontaneous activity. Any considerable change in spontaneous activity during the continuous recording was taken into account.

Temporal response strength was quantified for each response window following an odor stimulus by calculating the mean deviation (in frequency) from estimated spontaneous activity (MDS):

(1) $$\text{MDS}=\frac{1}{n}\overset{n}{\underset{i=1}{\sum }}|r_{i}-r_{\text{sp}}|$$

*n* is the number of bins, *r_i_* is the firing rate of bin *i* and *r*_sp_ is the estimated spontaneous activity. By taking the absolute value of the deviation, both positive and negative deviations contribute similarly to the MDS. This method enables quantification of complex responses consisting of both the inhibitory and excitatory elements without these neutralizing each other.

To ascertain the excitatory and inhibitory parts of the responses, the contribution of positive deviation (eMDS) and negative deviation (iMDS) was calculated. Mean response frequency (MRF) across bins of the response window and maximum frequency (maximum observed frequency in the response) was calculated as well. Odor responses were considered significant if either the excitatory or the inhibitory average MDS of two stimulations with the same odor exceeded the neuron’s standard deviation (SD) of spontaneous activity by a factor of two. To obtain the response profile curves of the neurons the significant responses were plotted over response window time. Responses to single odorants and mixtures of a neuron were determined as different, if their average MDS differed more than the pooled SD for all repetitions of the significant responses in that particular neuron. When the average MDS between the responses to mixtures and single odorant differed less than the pooled SD, the term hypoadditivity was used. If the single odorants MDS was stronger than the blend MDS, the term suppression was used. Hypoadditivity was only used to describe differences between responses to a blend and a particular single odorant, and not as a general mixture effect, because all constituents of the complex mixture were not tested. Neurons were sorted into three different groups: excitatory, inhibitory, and complex responding, depending on whether they responded significantly with eMDS, iMDS, or both in the response windows. Two-tailed Wilcoxon rank-sum tests were carried out on all significant responses in the population of selected neurons to compare the MDS response strength between the three different stimuli groups: control, selected single odorants, and blends. In order to represent the response kinetics, neuronal responses to selected odors were extracted. Binned frequencies for the defined response window were normalized with respect to either maximum firing frequency (excitatory neurons) or to spontaneous activity (inhibitory and complex neurons). Activity in each bin was color coded ranging from blue (inhibition) to red (excitation), with green being the range of spontaneous activity.

### Terminology

In naming the ACTs we followed the nomenclature proposed by Galizia and Rössler (2010) using terms according to the position in the brain. Thus, the inner, the medial, and the outer ACTs were in this study termed the medial (m), the medio-lateral (ml), and the lateral (l) APT, respectively. The term SP is according to Rø et al. (2007) the area proximately to and dorsally of the MB lobes, extending from the anterior to the posterior part. Concerning the anatomy of the MB lobes, we employ the system described in *H. virescens* by Rø et al. (2007). Here the α and α′ together with one arm of the γ lobe make up the vertical lobes and the second arm of the γ lobe and the β and β′ constitute the horizontal lobes. The heel (H) lays on the horizontal part anterior to the pedunculus. LP and LH denote to the areas defined in Kvello et al. (2009) and Løfaldli et al. (2010), i.e., LH is the small protrusion from the LP.

## Results

### Output areas of the antenno-protocerebral tracts

**Figure 1:**
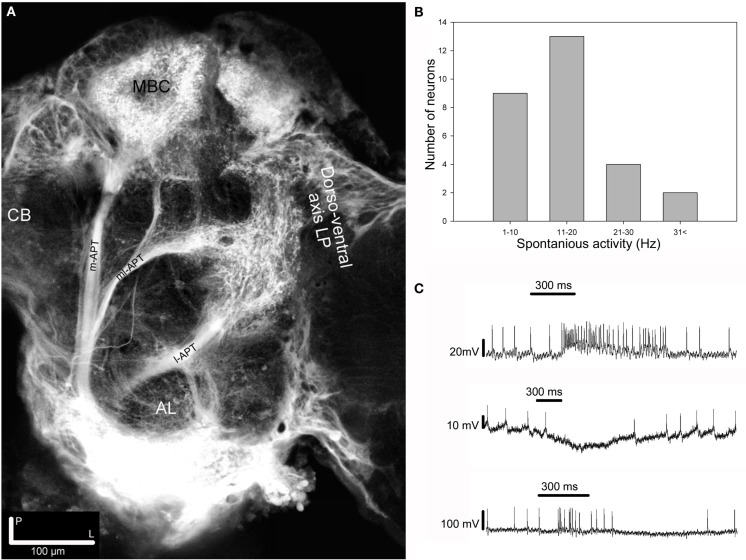
**Projection view from stacks of confocal images showing mass stained APTs, spontaneous frequency distribution and response modes among the 28 recorded neurons**. **(A)** Projection view from confocal images of mass staining in the AL revealing the three main APTs. The m- and the l-APTs projecting in the calyces of the MB and the OA of the LP, but in opposite order, and the ml-APT directly in the LP with some branches turning anterior-dorsally terminating in the SP. At this depth most m-APT projections in the LP are not visible. Dorsal view. CB, central body; MBC, mushroom body calyces; AL, antennal lobe; LP, lateral protocerebrum. **(B)** Histogram showing the frequency distribution of the spontaneous activity among recorded neurons in two target areas of the APTs, the SP, and the LP. **(C)** Intracellular recording showing the three response modes, excitation (upper trace), inhibition (middle trace), and complex (lower trace), elicited by stimulation with PB10 in three different neurons. All responses clearly outlast the stimulation period (300 ms).

Injection of dye in the AL of *H. virescens* revealed staining of the three major APTs, the medial, the medio-lateral, and the lateral (m-APT, ml-APT, and l-APT) and their target areas in protocerebrum (Figure 1A).

The calyces of the MBs clearly appeared with projections from the medial and the lateral tract. In the LP the dorso-ventral area of the OA received projections from all tracts. The third important olfactory area, the SP located dorsally of the posterior parts of the MB lobes, appeared with projections exclusively from the ml-APT.

### Electrophysiological recordings from protocerebral neurons

The presented results are based on recordings from 87 neurons obtained in the LP and in the SP. These neurons responded to stimulation with single odorants and blends containing the selected primary plant odorants (Table 1).

**Table 1:** **Overview of the most tested single primary odorants and blends in all of the 87 recorded olfactory neurons**.

Single odorants	Neurons tested (*n*)	Responses (%)	Blends	Neurons tested (*n*)	Responses (%)
Control	87	32	Plant blend 2	23	87
3Z-hexenyl acetate	43	61	Plant blend 3	12	66
Linalool	27	82	Plant blend 9	31	81
Ocimene	11	64	Plant blend 10	83	92
Geraniol	11	46	Plant blend 11	28	64
Germacrene D	38	82	Plant blend 12	50	96
Farnesene	11	46	
2-Phenyl ethanol	40	85	Air *P*(*r*): 0.32, singles *P*(*r*): 0.76, blends *P*(*r*): 0.93		

*The four single odorants and the two blends selected for further analysis as well as control are shaded. The weighted probability (*P*) to elicit responses is indicated for control, the four single odorants and the two blends (PB10 and PB12)*.

A puff of purified air (control) resulted in a weak response in about 32% of the neurons. The response probability varied between the primary plant odorants and the plant odor blends. The weighted average response probability (*P*) of the two most effective blends (*P* = 0.93, PB10 and PB12) was higher than for the four most effective single odorants (*P* = 0.76, 3Z-hexenyl acetate, linalool, 2-phenyl ethanol, and germacrene D; Table 1). The odorants constituting the six most tested plant odor blends are listed in Table 2.

**Table 2:** **Single odorants (shaded) constituting the six most tested blends, from the binary, PB2, to the most complex, PB12**.

Single odorants	Plant blends					
	PB2	PB3	PB9	PB10	PB11	PB12
3Z-hexanol						
3Z-hexen-1-ol						
3Z-hexenyl acetate						
Ocimene						
Linalool						
Geraniol						
(+)3-Carene						
E-verbenol						
Methyl benzoate						
2-Phenyl ethanol						
Farnesene						
Germacrene D						

The limited duration of the intracellular recordings, in emphasizing repetition as well as randomization of the stimuli, did not allow every neuron to be tested for all stimuli. We selected 28 of the 87 neurons for further response analysis, based on the required recording quality and the applied stimuli, as given in the method. Spontaneous activity in these neurons ranged from 1 to 42 Hz (Figure 1B). The identified response types to olfactory stimulation were excitation, inhibition, and complex responses, the latter consisting of inhibitory and excitatory phases (Figure 1C). Most responses outlasted the applied stimulus period. In the subsequent analyses of the 28 neurons the focus was on the responses to the following stimuli: Control, the four primary odorants, 3Z-hexenyl acetate, linalool, 2-phenyl ethanol, germacrene D and the two most complex plant odor blends, the 10 component PB10 and the 12 component PB12 (Table 1, shaded). PB12 contained all of the four mentioned odorants and PB10 all except germacrene D (Table 2).

The two blends and the four odorants showed highest response probability and was the most tested stimuli in all of the 87 recorded neurons. Twelve of the 28 selected neurons were tested for responses to stimulation with tastants. Three of them responded, one excitatory to sucrose and quinine applied to the left antenna (also excitatory responses to odors) and the two other to sucrose applied to the proboscis (one with excitatory and one with complex responses to odor stimuli). Occasional light and sound stimulation did not elicit responses in the 28 neurons.

### MDS and MRF

**Figure 2:**
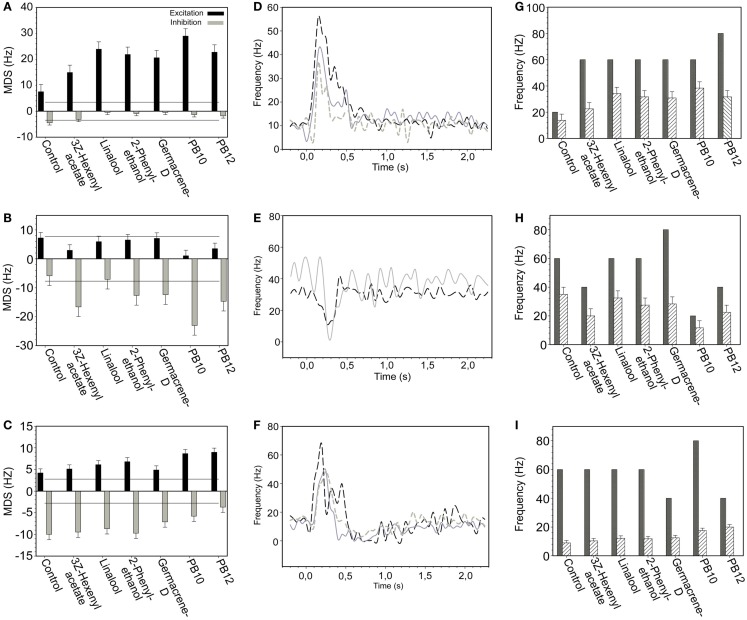
**Responses of selected neurons presented as MDS, MRF, and maximum frequency graphs with the response curves, exemplifying excitatory, inhibitory, and complex response modes**. **(A–C)** Average MDS plot for control, four single odorants and the two blends, PB10 and PB12 of **(A)** an excitatory, **(B)** an inhibitory, and **(C)** a complex responding neuron. In **(A)** only the response in the excitatory phase is significant (black bars) and in **(B)** only in the inhibitory phase (gray bars). Lines indicate significant response threshold. **(D–F)** The response curves for all response modes clearly reflect the corresponding MDS plot. **(D)** Response curves for three short excitatory, **(E)** for two short inhibitory, and **(F)** for three complex responding neurons. **(G–I)** Responses presented as maximum frequency (black bars, first response only) and MRF (striped bars) for the same neurons as in **(A–C)**. Evidently the mean and the maximum frequency for the inhibitory **(H)** and the complex **(I)** responding neurons do not reflect the response curve.

The average responses of the 28 neurons to odor stimuli was calculated as MDS indicating response strengths (both excitatory and inhibitory), MRF, and maximum frequency (Figures 2A–I).

Neurons were further grouped according to their MDS response properties as exemplified in Figures 2A–C. The MDS response property of the individual neurons (Figures 2A–C) is also reflected in the response profile curve (binned firing rate) of the neurons in Figures 2D–F. All neurons assigned to the group of excitatory and inhibitory responses, showed positive or negative MDS, respectively, in the main part of the response window (Figures 2A,B,D,E). In a few cases a transient change of the response appeared as exemplified by the MDS (Figure 2B) and the dotted curve (Figure 2E) showing a weak excitation following the inhibitory responses. This kind of changes was rarely significant. In the complex responding neurons exemplified in Figures 2C,F, a strong inhibitory response phase followed a stronger excitatory phase. These transient changes of response phases are obviously not reflected in the MRF (Figure 2I).

**Figure 3:**
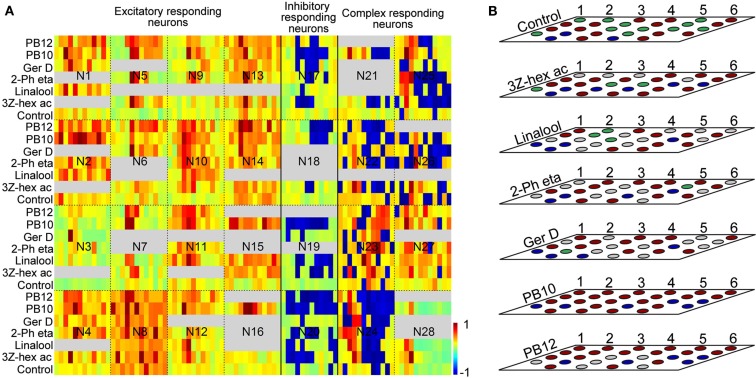
**(A)** Kinetics of recorded responses of all 28 neurons to the selected stimuli: control, 3Z-hexenyl acetate, linalool, 2-phenyl ethanol, germacrene D, and the mixtures PB10 and PB12. Single trace responses are displayed as binned firing rate in color codes normalized with respect to maximum spike rates for the excitatory [neuron (N) 1–16] and spontaneous activity for the inhibitory (N17–N20) and the complex responding neurons (N21–N27). N28 is the unassigned neuron. Firing activities range from −1 (dark blue = maximum inhibition) to 1 (dark red = maximum excitation). All response windows are defined by 12 bins with sizes: 200 ms (for N19 and N20), 100 ms (N15, N16, N21, N24, and N25), and 50 ms for the remaining neurons in the figure. Gray fields indicate untested stimuli. **(B)** Response modes based on significant MDS of the same 28 neurons as in **(A)** to the selected stimuli. The neurons are numbered as indicated from left to the right, starting with the upper line. Each layer represents the same neurons for different stimuli which are sorted according to response modes: Neurons N1–N16 as excitatory, N17–N20 as inhibitory, and N21–N27 as complex responding and neuron 28 is unassigned. For the complex responding neurons the strongest response phase is indicated. Red: excitation, blue: inhibition, gray: not tested, green: no response. 3Z-hex ac, 3Z-hexenyl acetate; 2-Ph eta, 2-phenyl ethanol; Ger D, germacrene D.

Response kinetics represented as normalized color coded firing rate levels at different time bins for the 28 neurons (Figure 3A) support the grouping of neurons based on MDS levels shown in Figures 2A–C and Figure 3B.

Sixteen neurons (N1–N16 in Figures 3A,B) appeared with clear excitatory response profiles to odor stimuli, four neurons (N17–N20 in Figures 3A,B) mainly with inhibitory responses and seven neurons (N21–N27 in Figures 3A,B) with a complex response pattern of both excitatory and inhibitory phases. Neuron N28 in Figures 3A,B was unassigned because of close to zero spontaneous activity and a particular response pattern of two excitatory phases separated by a long inter-response interval of several seconds (only the first excitatory phase visible in Figure 3A). The response duration of the excitatory responding neurons varied, 14 showing short responses (150–600 ms) and 2 longer responses (1200–2400 ms). The latency also differed, being 100–200 ms in the former and 250–350 ms in the latter group. Among the four inhibitory responding neurons, two showed short lasting (400–600 ms) and two longer lasting (1200–1800 ms) responses. Neurons placed in the complex responding group showed latency between 150 and 300 ms and response duration between 400 and 1100 ms. For comparison of response kinetics, the bin sizes in Figure 3A were adjusted according to the response duration, varying between 50 ms bins for the 600 ms responses, 100 ms bins for the 1200 ms responses, and 200 ms bins for the 2400 ms responses. For the response to 2-phenyl ethanol in N9, displayed in Figure 3A, the MDS significance is not indicated in Figure 3B because of a lack of repetition for this stimulus. Analysis of the response kinetics revealed in the majority of neurons overlap of the temporal response patterns for different odor stimuli, with maximum or minimum firing rates in the same or adjacent time bins (Figure 3A). However, variation in firing rate and decay of the responses for the different stimuli appeared with a general tendency of larger similarity between the two mixtures and between the single odorants than between mixtures and single odorants.

The response modes of all 28 neurons given in Figure 3B showed more frequently excitatory than inhibitory responses for all stimuli, also applying to the control (excitation in 14, no response in 11, and inhibition in 3). The control stimulus elicited responses in 60% of the 28 neurons, somewhat more frequently than among all 87 neurons (Table 1). The two mixtures PB10 and PB12 was the most potent stimuli, PB10 elicited responses in all 28 neurons and in 92% of totally tested 83 neurons and PB12 in all 22 tested among the 28 neurons and in 96% of 50 tested neurons (Table 1). All neurons responded with significant MDS to PB10 and to at least one single odorant, with exception of two neurons (N15 and N16 in Figure 3B, the latter neuron tested for only one single odorant) that showed significant responses exclusively to PB10 (Figure 3B).

### Odor discrimination expressed by response strength

**Figure 4:**
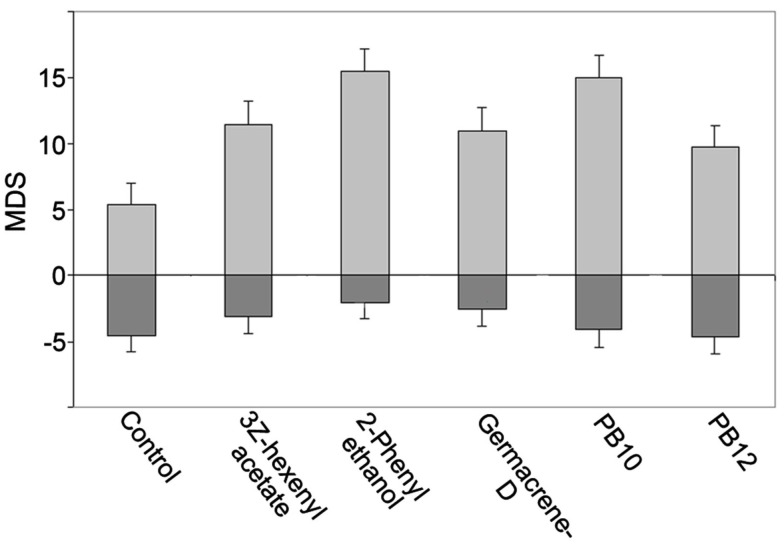
**Responses presented as MDS bar plot for an excitatory responding neuron revealing an intricate response pattern to the single odorants and the blends**. The response to PB10 showing hypoadditivity as compared to the 2-phenyl ethanol response. However, addition of two components (PB12) resulted in a weaker response (suppression), but hypoadditivity in comparison to the responses to 3Z-hexenyl acetate and germacrene D. Light gray bars, excitatory MDS; Dark gray bars, inhibitory MDS (not significant). Error bars indicate pooled SD.

A comparison of the odor responses revealed different MDS and MRF in most of the 28 neurons to the control, the single odorants, and the mixtures, as exemplified in Figures 2A–C,G–I. In the 16 neurons with excitatory responses, stimulation with the blends yielded the strongest responses (largest MDS and the highest MRF) in 11 of them (PB10 strongest in six and PB12 in five neurons, two of them, N15 and N16 in Figures 3A,B with long lasting excitation were only tested with PB10). Suppression was observed in two neurons for which a single odorant (linalool and 2-phenyl ethanol, respectively) yielded stronger MDS than the blends, PB10 and PB12. Hypoadditivity was seen in three neurons for which the strongest single odorant and the strongest blend yielded equal responses among the tested odorants. A more intricate relation was often found when comparing the responses to single odorants and the blends (Figures 2A–C; Figure 4).

**Figure 5:**
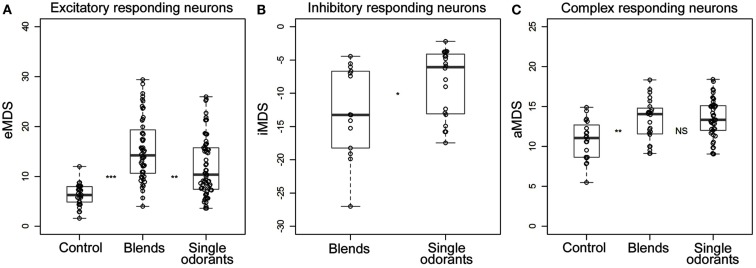
**All significant responses of (A) excitatory, (B) inhibitory, and (C) complex responding neurons selected and represented in box plots**. The circles indicate the calculated MDS values (Hz) for the individual responses to the control, single odorants, and the complex blends. For the excitatory neurons only the positive deviation (excitatory MDS) was considered, for the inhibitory neurons only the negative deviation (inhibitory MDS) and for the complex responding neurons the absolute deviation. Wilcoxon rank-sum tests were used to compare the observed values between the three stimuli groups (control, the four single odorants and the two blends selected for analysis). Asterisks indicate significance levels of the tests (*→*p* < 0.05, **→*p* < 0.01, ***→*p* < 0.001). Since only one inhibitory neuron responded to control, this was not included in the statistical analysis.

In the neuron in Figure 4 the response to the strongest blend (PB10) and the strongest single odorant (2-phenyl ethanol) was about equal, implying hypoadditivity. However, in spite of the two added odorants, germacrene D and farnesenes, PB12 showed suppression in relation to 2-phenyl ethanol, but hypoadditivity in relation to germacrene D and 3Z-hexenyl acetate. All responses were clearly stronger than to the control. Different response strength to the single odorants was found in most neurons between the strongest and the weakest odorants and only in four neurons between the two strongest single odorants. The statistical analysis of the grouped MDS strength in the excitatory responding neurons (Figure 5A) showed significant stronger responses to the odorants than to control (air vs. singles *p* = 1.14E−06 and air vs. blends *p* = 4.52E−11) and significant stronger responses to blends than to single odorants (*p* = 3.11E−03).

Among all 16 excitatory neurons the highest observed MDS value (29 Hz) and MRF (39 Hz) was obtained for PB10. The highest observed maximum response frequency (140 Hz) was elicited by stimulation with 3Z-hexenyl acetate, 2-phenyl ethanol, and PB12 in one neuron. The inhibitory responding neurons also displayed different MDS strength in their responses to stimulation with the single odorants and mixtures. Two neurons responded stronger to PB10 than to any of the other odorants (only one of them tested with PB12). The other two inhibitory responding neurons showed hypoadditivity and did not differ in response strength to the strongest single odorant and the strongest mixture. The control elicited response in one neuron, which was clearly weaker than the responses to odor stimulation. The statistical tests of grouped MDS strength showed stronger responses to the blends than to the single odorants in the neurons responding by inhibition (*p* = 0.02997; Figure 5B).

In the complex responding neurons the effect of the single odorants and mixtures varied more than in the two previously described groups (Figure 5C). All neurons showed either suppression or hypoadditivity in one or both of the response phases. Four of them showed suppression; one in both phases, two in the inhibitory and one in the excitatory phase and three of them showed hypoadditivity; two in both phases and one in the excitatory phase. Discrimination between the two complex mixtures and between the two strongest single odorants also varied across neurons as well as within a neuron. Four neurons showed stronger MDS to one of the two mixtures (two to PB10 in the excitatory phase and two to PB12 in the inhibitory phase) and two neurons showed different MDS to the two strongest single odorants (one neuron in the inhibitory phase and the other in the excitatory phase). Statistical analysis of the absolute MDS values in complex responding neurons revealed that single odorants (*p* = 0.000189) and blends (*p* = 0.004335) elicited significantly stronger responses than the control (Figure 5C). Although the difference in MDS strength between single odorants and blends was not significant, the data pointed toward a stronger absolute MDS for the single odorants than for the blends. In the unassigned neuron MDS analysis on the first response phase revealed strongest excitatory response to 3Z-hexenyl acetate, whereas PB10 and linalool elicited similar responses as control. This neuron was bilateral, having dendritic arborizations in the right LP and axonal projections in the OA of the left LP.

### Neurons innervating olfactory areas in the protocerebrum

**Figure 6:**
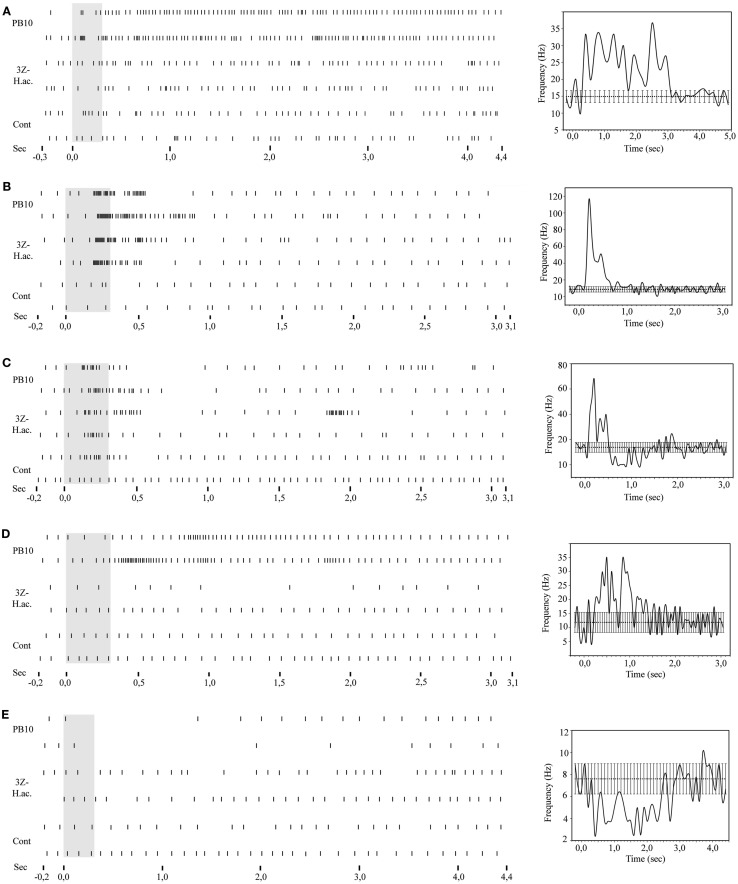
**Raster plots and response curves of stained neurons responding to stimulation with the PB10 blend**. **(A–E)** Left: the raster plots include responses to the same stimuli (control, 3Z-hexenyl acetate, and PB10) for the five stained neurons, **(A)** the Type 1 ml-APT PN, **(B)** the MB-SP neuron, **(C)** the MB-LP neuron, **(D)** the LP-SP neuron, and **(E)** the LP-descending neuron. Right: corresponding response curves showing the average temporal response pattern. 3Z-hex ac, 3Z-hexenyl acetate; Cont, control.

Nine of the 28 neurons were fully stained, showing innervation in the three main olfactory protocerebral areas, the SP, the LP, and the MB. The focus here is on five neurons, all responding to the blend PB10 (Figures 6A–E).

**Figure 7:**
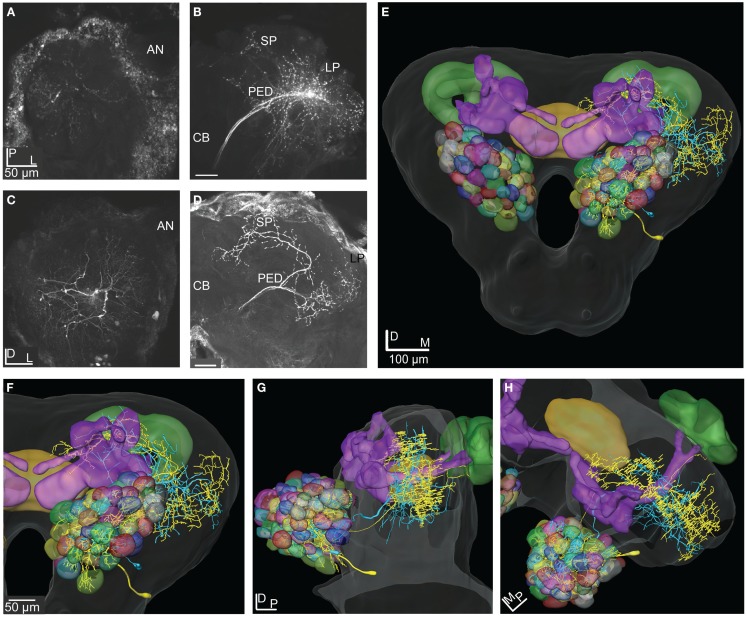
**Projection view from stacks of confocal images of two types of ml-APT neurons, 3D reconstructions and transformations into the SBA**. **(A)** Confocal images of the two simultaneously stained Type 1 ml-APT PN showing the dendritic arborization in the AL with spars innervation in many glomeruli. **(B)** The axonal branches projecting in the two protocerebral areas, the SP (sparsely) and the LP. **(C)** Confocal images of the stained Type 2 ml-APT PN showing loose dendritic arborization in most or all glomeruli. **(D)** Axonal projections of the same neuron showing extensive and wide branching patterns in the SP and the LP. One axon branch off before reaching the LP and making the characteristic dorso-anterior turn before entering the SP. **(E–H)** 3D reconstructions of the ml-APT PNs (Type 1; blue and Type 2; yellow) transformed into the SBA showing extensive overlap in the OA of the LP, both axons branching in a dorsal and ventral direction. In the SP some axonal projections are proximate to the MB lobes. Both neurons had soma ventrally in the lateral cell cluster of the AL. **(E)** Frontal view, **(F)** close-up frontal view, **(G)** lateral view, and **(H)** dorsal view. AN, antennal nerve; PED, pedunculus; CB, central body; LP, lateral protocerebrum; SP, superior protocerebrum.

**Figure 8:**
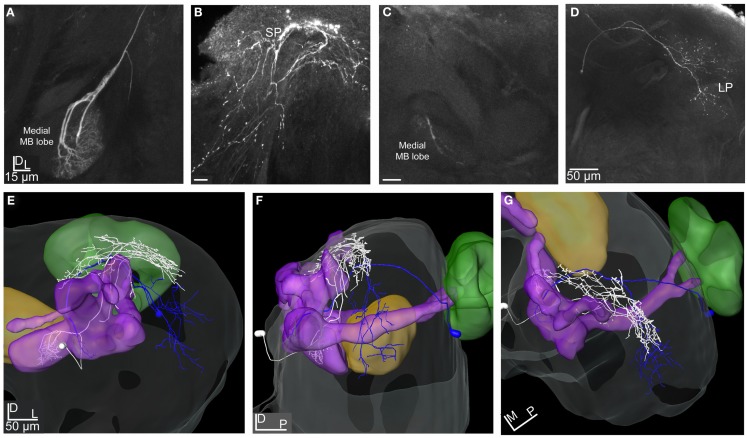
**Projection view from stacks of confocal images and 3D reconstruction of two stained efferent MB extrinsic neurons transformed into the SBA**. **(A)** Projection view of the MB-SP neuron showing the dense dendritic arborization in the medial lobe and **(B)** the relatively wide axonal projections in the SP proximate to the vertical lobe. **(C)** Confocal images of the MB-LP extrinsic neuron showing the weak dendritic innervation in the lateral part of the medial lobe and **(D)** the axonal projection in parts of the OA of the LP. **(E–G)** 3D reconstruction of the MB-SP (white) and the MB-LP (dark blue) transformed into the SBA visualize the different axonal projections in the SP and LP. **(E)** Frontal view, **(F)** lateral view, and **(G)** dorsal view. SP, superior protocerebrum; LP, lateral protocerebrum; MB, mushroom bodies.

**Figure 9:**
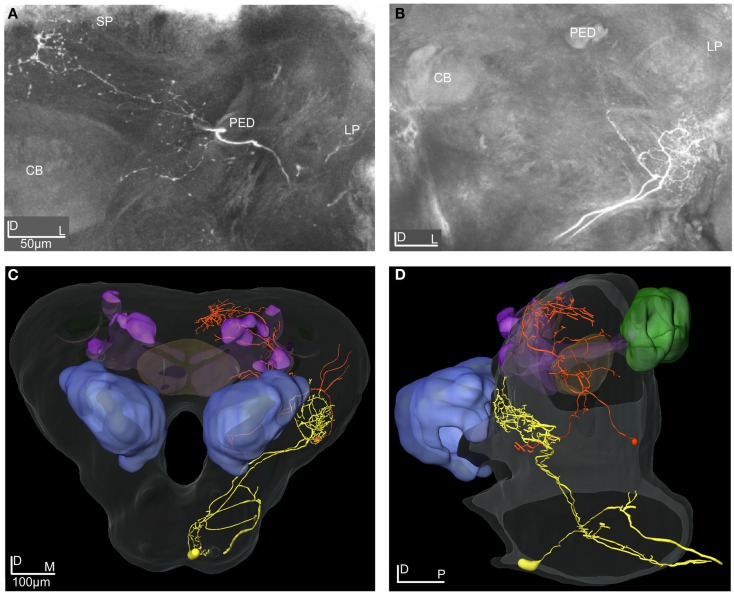
**Projection view from stacks of confocal images of the LP-SP and the LP-descending neuron reconstructed and transformed into the SBA**. **(A)** Projection view of the LP-SP neuron showing dendritic arborizations in the LP and axonal projections in the SP. **(B)** Projection view of the LP-descending neuron having two dendritic branches originating in the SOG and terminating in dense arborizations in the ventro-anterior area of the LP. **(C,D)** 3D reconstructions of the two neurons transformed into the SBA visualizing the dendritic arborizations and the axonal projections of the LP-descending neuron (yellow), the axon leaving the brain through the ipsilateral connective. The large cell soma is located medio-ventrally in the SOG. The LP-SP neuron (orange) has a few dendritic branches in the OA of the LP and in the medio-inferior parts of the protocerebrum. The dense axonal projections are medially in the SP and some branches terminate around the lobes. No overlap was found between the LP-SP and the LP-descending neuron. **(C)** Frontal view and **(D)** lateral view. CB, central body; PED, pedunculus; SP, superior protocerebrum; LP, lateral protocerebrum.

**Figure 10:**
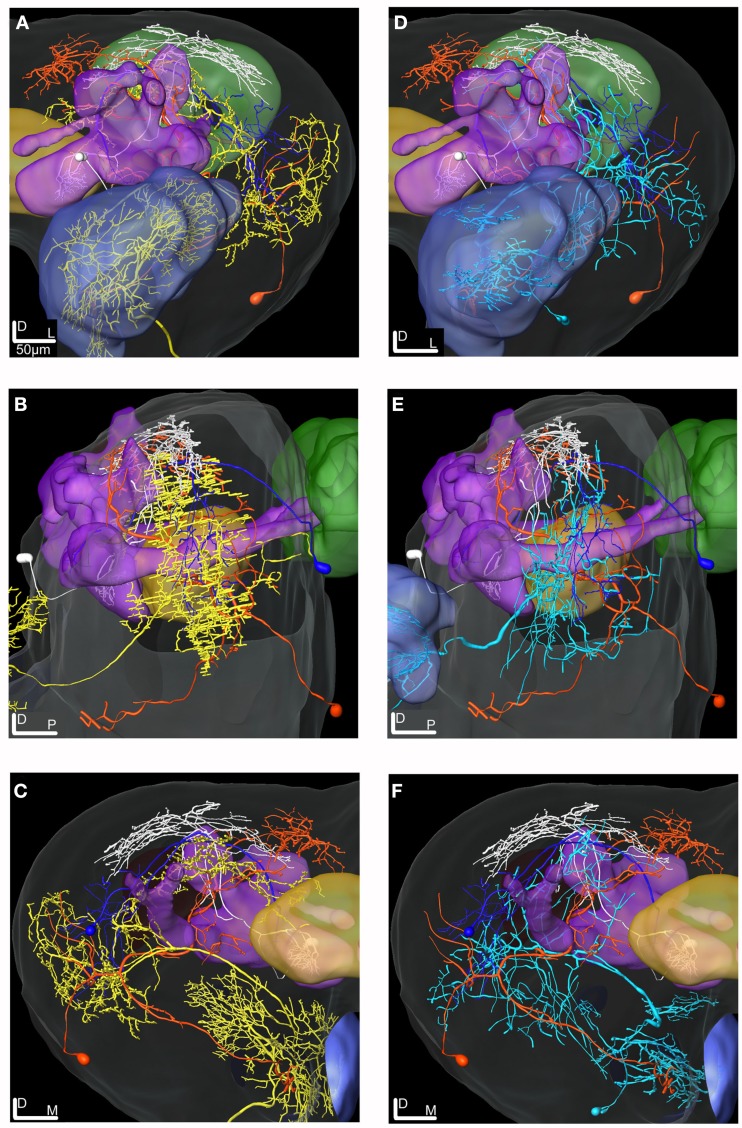
**3D reconstructions of the ml-APT PNs, the MB extrinsic neurons and the LP-SP neuron transformed into the SBA**. **(A–C)** Type 2 ml-APT PN (yellow) shows overlap in the OA of the LP with both the dendrites of the LP-SP (orange) neuron and the axonal projections from the MB-LP neuron (dark blue). Three neurons, the ml-APT, the MB-SP (white), and the LP-SP, having partly overlapping axonal terminals in the SP, indicate input from multiple brain areas. The MB-SP neuron has most terminals in a more dorsal part of the SP than the two other neurons. **(A)** Frontal view, **(B)** lateral view, and **(C)** posterior view. **(D–F)** The Type 1 ml-APT (blue) neurons (two simultaneously stained) transformed into the SBA together with the MB extrinsic neurons and the LP-SP neuron. Due to the sparser projection pattern of the Type 1 ml-APT PN a less extensive overlap with MB-LP axonal terminals and the LP-SP dendrites appeared. **(D)** Frontal view, **(E)** lateral view, and **(F)** posterior view.

**Figure 11:**
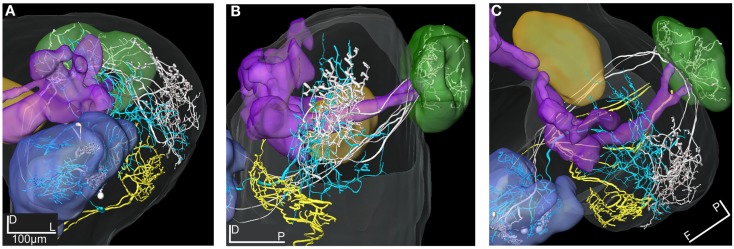
**3D reconstructions of two PN types and the LP-descending neuron visualized in the SBA**. **(A–C)** The projections of the three m-APT PNs (white) are most dorsal in the LP, clearly separated from the more ventral dendrites of the descending neuron (yellow). The wide projections of the ml-APT neurons (blue) partly overlaps with the m-APT PN projections and extend close to the area of the descending neuron dendrites. **(A)** Frontal view, **(B)** lateral view, and **(C)** dorsal view.

One neuron was an ml-APT PN showing similar morphology as another neuron stained by electroporation, but not physiologically studied. The other four neurons inspected in confocal images and 3D reconstructions were protocerebral neurons with projections in partly overlapping areas in the SP and the OA of LP (Figures 7–11). All neurons were transformed into the SBA.

### Medio-lateral APT projection neurons

The two stained ml-APT PNs revealed different but partly overlapping axonal projections as well as different glomerular innervation patterns in the AL (Figures 7A–H). Both were multiglomerular, the Type 1 having a sparsely dendritic arborization in each of the innervated glomeruli, most of them located laterally and medially in the AL (Figures 7A,B,E–H, two Type 1 neurons stained during the same recording, blue reconstruction). The Type 2 neurons (*n* = 2, only one shown in Figures 7C–H, yellow reconstruction) innervated densely almost all glomeruli of the AL. All four PNs had soma in the ventral part of the lateral cell cluster. The axons of the Type 1 ml-APT PNs projected in the medial part of the dorso-ventral OA in the LP, with some overlap with the more dorsal projections of m-APT PNs (Figures 11A–C, m-APT PNs from Løfaldli et al., 2010, termed IACT PNs). A few branches of the ml-APT PNs extended into the LH and two others posteriorly toward the ipsilateral calyces. Some side branches turned more dorso-medially from the LP into to the SP. Here they branched off closely to the MB lobes and the pedunculus, dorsally, and posteriorly to the α, α′ (vertical lobes) and the γ lobe, the heel, and pedunculus. The axons of Type 2 ml-APT PNs divided after passing ventro-laterally to the pedunculus (Figures 7D–H, only one included). One branch turned dorso-medially into SP where it showed extensive arborizations proximately to the MB lobes, partly overlapping with the Type 1 ml-APT PNs. A few smaller branches extended more dorso-medially and posteriorly to the medial part of β′, β (medial lobes), and γ lobes (Figures 7E–H, only one showed). The second major branch ran laterally into the dorso-ventral axis of LP overlapping with the projections of Type 1 ml-APT PNs (Figures 7E–H). One single projection ran along and within the outer part of the pedunculus in posterior direction toward the ipsilateral calyces where it branched off in a confined area external to the calyces. Raster plot and the response curve from the recording of one Type 1 ml-APT PN is shown in Figure 6A. The responses appeared as a long lasting excitation to stimulation with PB10 showing a latency around 300 ms. No responses were recorded to stimulation with control, other mixtures (B5 and ylang oil) or single odorants (3Z-hexenyl acetate, linalool, geraniol. Neuron number N15 in Figures 3A,B). In the recording of one of the Type 2 ml-APT PN no response was obtained to stimulation with control, single odorants, or blends (not included among the 28 physiologically described neurons in this study).

### Mushroom body extrinsic neurons

Two MB extrinsic neurons were reconstructed and transformed into the SBA (Figures 8A–G). Both neurons had their dendritic arborizations within the medial lobe of the MB. One, named MB-SP extrinsic neuron (Figures 8A,B,E–G, white reconstruction), showed dense arborizations in a confined area of the swelling part of the lobe, whereas the other extrinsic neuron (MB-LP) had a sparse pattern with only one dendritic branch within the lobe (Figures 8C–G, blue reconstruction). The soma of the MB-SP extrinsic neuron was located frontally of the MB lobes and of the MB-LP extrinsic neuron posteriorly and closely to the MB calyces. As indicated by the name, the MB-SP extrinsic neuron had its axonal projections located in the SP with branches posteriorly and dorsally to the α, α′ (vertical), and the γ lobe. Some branches extended more laterally terminating in a dense projection pattern (Figures 8E–G). The axonal projections of this extrinsic MB neuron partly overlapped with the axons of both types of ml-APT PNs in the SP (Figures 10A–F). This implies that SP in *H. virescens* receives input from both types of neurons in the same or partly overlapping areas. The stained MB-LP extrinsic neuron projected in the OA of LP, with relatively sparse branches extending from dorso-medial to more ventro-lateral areas (Figures 8D–G). The projections partly overlapped in the LP with the axonal projections of both types of ml-APT PNs (Figures 10A–F). This implies that LP like the SP in *H. virescens* receives information from the MB lobes as well as from the AL.

Raster plots for the MB-SP extrinsic neuron (Figure 6B) and for the MB-LP extrinsic neuron (Figure 6C) show a strong response of both neurons to stimulation with PB10. While the responses of the MB-SP extrinsic neuron appeared as a clear excitation, the responses of the MB-LP extrinsic neuron were typically complex (Figures 6B,C, respectively). The MB-LP neuron showed a weak response to control and a slightly stronger excitation to stimulation with the mixture PB10 (PB12 not tested) than with the single odorant 3Z-hexenyl acetate eliciting a slightly stronger inhibitory than excitatory phase (neuron number N21 in Figures 3A,B). The temporal response pattern clearly differed for the blend and the single odorant (Figure 6C). The excitatory phase of the first response to PB10 was clearly stronger and longer lasting than of the repeated response. The opposite pattern appeared for the single odorant. The maximum frequency was also different between the responses to the blend (100 and 80 Hz) than to the single odorant (80 and 60 Hz). This neuron did not respond to stimulation with tastants, which were applied after odor stimulation in order to avoid influence on odor responses. In the LP-SP neuron the responses to stimulation with the single odorants and the blends showed different strength, clearly strongest to the blends PB10 and PB12, weaker to 3Z-hexenyl acetate, weakest to 2-phenyl ethanol, and no response to control. The difference between the PB10 and PB12 responses was too small to be considered as significant. Like the MB-LP neuron, the MB-SP neuron displayed a stronger response to the first than to the repeated stimulation with the blends and the opposite pattern for 3Z-hexenyl acetate (Figure 6B, neuron number N5 in Figures 3A,B). The measured latency in the two MB extrinsic neurons was between 150 and 200 ms.

### Lateral protocerebral neurons

The two reconstructed and transformed lateral protocerebral neurons differed in respect to dendritic arborizations and axonal projections (Figures 9A–D). Both neurons had their dendrites in the LP but different axonal projections, one in the SP (LP-SP neuron), and the other projecting in the ipsilateral connective (LP-descending neuron). The dendritic arborization of the LP-SP neuron was sparse in the LP with a few branches extending toward the central body and terminating proximate but outside (Figures 9A,C,D, orange reconstruction). The dendrites of the LP-descending neuron showed a dense and more confined pattern located ventrally in the LP with the terminals arranged in an anterior-posterior direction (Figures 9B–D, yellow reconstruction). The dendritic arborizations were close but not directly overlapping with the LP-SP dendrites (Figures 9C,D) or with the axonal projections of the Type 1 ml-APT PNs (Figures 11A–C). After registration of the neurons into the standard atlas it became evident that the dendrites of the LP-descending neuron were positioned anterior-ventrally to the OA of the LP (Figures 11A–C, m-APT PNs from Løfaldli et al., 2010). Comparison of the confocal images of the LP-descending neuron and the mass stained APTs (Figure 1A) indicated that the descending neuron dendrites might have a direct overlap with some of the most ventral PN projections of the ml-APT or the l-APT. The LP-descending neuron, having a large soma in the SOG, appeared with two major dendritic branches extending from the SOG to the LP and giving off a few small branches in the SOG before the axon was leaving the brain via the ipsilateral connective. Unfortunately, the connectives and the thoracic ganglion were not dissected out for further investigations of the axonal projections (Figures 9B–D). The axon of the LP-SP neuron projected dorso-medially from the LP into the SP. Here it ramified relatively densely posterior and medial to the α, α′, and γ lobe (vertical lobes) with a few branches projecting slightly more ventrally. The cell soma was located laterally on the posterior side of the brain (Figures 9A,C,D). Some of the axonal projections of the LP-SP neuron showed partly overlap with axonal projections of both ml-APT PN types and of the MB-SP extrinsic neuron (Figures 10A–F).

The LP-SP neuron and the LP-descending neuron both responded to PB10, but differently, as shown by the raster plot and the response profile curves in Figures 6D,E, respectively. The LP-SP neuron showed a long lasting excitation consisting of two peaks, as seen in the response to the first stimulation with the blend (Figure 6D). No response was obtained to stimulation with 3Z-hexenyl acetate and the control (neuron number N16 in Figures 3A,B). Unfortunately, other odorants were not tested. The LP-descending neuron responded with a long lasting inhibition, the longest to the first stimulation with PB10 (raster plot Figure 6E). A considerably weaker inhibition was recorded to stimulation with other blends (PB2, PB3, and PB4) and to the first stimulations with linalool and germacrene D (neuron number N19 in Figures 3A,B). The other tested odorants (3Z-hexenyl acetate, E-verbenol, farnesenes) and control did not elicit any response. Measured latency for the LP-SP neuron was around 250 ms and around 300 ms for the LP-descending neuron.

### The putative circuit

The two MB extrinsic neurons receiving information in the medial lobe and projecting in the LP and the SP, respectively, had partly overlapping axonal projections with the ml-APT neurons, as shown after transformation into the standard atlas (Figures 10A–F). In the LP the axonal projections of the ml-APT neurons and the MB-LP neuron overlapped with the dendritic arborizations of one lateral protocerebral neuron, LP-SP, having axonal projections in the SP. In the SP the axonal projections of the LP-SP neuron overlapped with the projections of the other axonal branch of the ml-APT neurons as well as with the axonal projection of one of the MB extrinsic neurons. Thus, these neurons might form a circuit where the SP directly receives information from the AL via the multiglomerular ml-APT neurons and indirectly via the MB extrinsic neuron, as well as from the LP. The input to the LP in the dorso-medal part is originating in the AL, via all three APTs, as well as in the MB. Output from protocerebrum was only found by the LP-descending neuron.

## Discussion

Based on intracellular recordings from SP and LP, we have described neurons involved in a putative circuit processing plant odor information in the brain of *H. virescens* females, 28 neurons presented in this study. We have stimulated with primary odorants, each identified as the best odorant for a particular ORN. Thus, we know that the responses obtained in the central neurons to stimulation with the single primary odorants and mixtures of them contain relevant information that can be ascribed to particular, functionally described ORN types. The response profiles, classified as pure excitation, pure inhibition as well as mixed excitation-inhibition, are similar to responses described for other central olfactory neurons, like the AL PNs of several insect species, including heliothine moths (Christensen et al., 1991, 1995; Vickers et al., 1998; Heinbockel et al., 1999; Barrozo et al., 2011; Kuebler et al., 2011). Compared to the sparsely obtained responses to plant odorants in previous recordings from the AL of *H. virescens* (unpublished), we obtained more frequently responses when recording from the SP and the LP. Most olfactory neurons responded to several of the primary odorants and the blends and only a few showed more specific responses to one or two odorants. This implies that the information about different primary odorants to a large extend is integrated in these higher order protocerebral neurons. In contrast, most of the medial tract AL PNs, the majority of them innervating a single and fewer two-three closely located glomeruli, as shown in *H. virescens* and *Bombyx mori*, may get excitatory input from one or two-three types of ORNs (Løfaldli et al., 2010; Namiki and Kanzaki, 2011). Thus, when recording from the AL in contrast to protocerebrum, there is a lower probability to stimulate with the particular primary odorant, which explains the sparse responses obtained in *H. virescens*. Furthermore, sparse responses to single odorants may also apply to multiglomerular PNs as shown in this study for the Type 1 ml-APT PN, exclusively responding to the PB10 blend (Figures 6A and 7A,B,E–H). Local excitatory interneurons have not been found in this or in other moth species, in contrast to the fruit fly where intraglomerular excitation is shown (Olsen et al., 2007; Shang et al., 2007), which may contribute to a broader response profile of the PNs (Wilson et al., 2004).

To understand how odor information is integrated in the central neurons of *H. virescens*, we have compared the responses elicited by single primary odorants and two complex mixtures containing the single odorants. In these comparisons we have used the MDS which quantifies response strength as mean deviation from spontaneous activity in the temporal phases of the response window. We are aware of the limitation of the results. Due to the relative short duration of this kind of intracellular recordings every neuron was not tested for all odorants in the protocol. However, from the data on the 28 selected neurons tested for the most effective odorants and mixture, several integration principles appeared, like hypoadditivity in 8 neurons, suppression in 6 neurons, and best mixture effect in 16 neurons. Because hypoadditivity and best mixture effect would require tests with all single constituents, we cannot conclude whether the responses were exclusively due to a mixture effect and not to another single constituent that was not tested. In spite of these limitations, the results show that the integration is more intricate than just hypoadditivity and suppression, as exemplified in the results (Figures 2A–C and 4). Thus, a neuron might show similar response strength to a mixture (PB10) and to one of the single odorants, but the addition of two other excitatory odorants to the same mixture (PB12) elicited a weaker response. Most likely the integrating neurons are activated by an array of input channels which might contribute with different strengths to the evoked post synaptic responses when activated alone by single odorants or in concert by mixtures. In addition to the complex integration in some neurons, others showed more specific responses to stimulation with one or two of the tested odorants.

### Putative circuit

Based on the physiology and morphology of the successfully stained neurons presented in this study, we consider them as part of a putative circuit receiving input from the AL and connecting the three protocerebral areas, the SP, the LP, and the MB. Anatomical overlap does not automatically indicate functional connectivity. However, the responses by all of them to stimulation with the 10 component mixture indicate that they are part of a putative circuit involved in processing information given by this blend. Like in other insect species, the calyces of the MB and the LP in *H. virescens* are the targets of the m- and l-APT neurons, but in the opposite order (Rø et al., 2007; Galizia and Rössler, 2010). The protocerebral olfactory areas, the calyces, the SP, and the LP in *H. virescens* are visualized by the mass stained APTs (Figure 1A), the calyces, and the LP covering the projection area of the three m-APT neurons, as shown in Figures 11A–C (m-APT PNs from Løfaldli et al., 2010). The typical five axonal branches of the m-APT neurons innervating the calyces appear before the axon run anterior-laterally into the OA of the LP, as previously shown in Rø et al. (2007) and Løfaldli et al. (2010).

### The ml-APT neurons

The mass stained ml-APT (Figure 1A) with the distinct pathways and projections in the SP and the LP of *H. virescens*, show slightly different axonal branching pattern than ml-APTs described in other insect species, like in the honeybee (Kirschner et al., 2006; Galizia and Rössler, 2010). Whereas SP is one target area of some ml-APT neurons in *H. virescens* (Figures 1A and 7B,D,E–H) the ring neuropil around the α-lobe of the MB seems to be a corresponding area in the honeybee (Abel et al., 2001; Kirschner et al., 2006). However, the multiglomerular innervation by ml-APT PNs is typical (Abel et al., 2001; Jefferis et al., 2007; Rø et al., 2007; Galizia and Rössler, 2010; Namiki and Kanzaki, 2011) as well as the presence of GABAergic PNs in this tract, as shown in several species (Hoskins et al., 1986; Schäfer and Bicker, 1986; Berg et al., 2009). The morphology of the identified ml-APT PNs of *H. virescens* in this study (Figures 7A–H) and in Rø et al. (2007) shows similarities by multiglomerular, but sparse innervation and projections in the LP and SP, as well as heterogeneity in respect to wide or condensed projection patterns and axonal pathways with distinct bifurcation in only some of them. Similar morphological patterns of two ml-APT neurons have previously been described in the honeybee (Abel et al., 2001).

The physiological responses recorded in the Type 1 ml-APT neuron (Figure 6A) seem to reflect the sparse dendritic arborization in each glomerulus (Figures 7A,E–H). Since the neuron responded with strong excitation and long latency exclusively to the complex mixture of 10 primary odorants and showed no responses to the single odorants in this mixture or to other less complex blends, it implies that the neuron needs input from multiple glomeruli in concert to become activated. This is in contrast to neurons responding to single odorants and having dense innervation in one glomerulus. The correlation between innervation pattern and physiology of multiglomerular neurons has been a matter of speculations in earlier morphological studies by Kirschner et al. (2006) *citation*: “Due to the rather sparse dendritic innervation within glomeruli these neurons probably have a high activation threshold.” The functional implication of the ml-APT neurons in *H. virescens* might be to transfer information of specific activity patterns from the AL to the target regions in SP and LP. Different multiglomerular ml-APT neurons might display functional heterogeneity. In the honeybee responses to single odorants by ml-APT neurons have been shown (Abel et al., 2001). The ml-APT seems to contain both excitatory and an inhibitory channels in parallel to the excitatory pathways formed by the m- and the l-APT PNs, indicating integration of information in third order LP neurons. Possibly, GABAergic ml-APT neurons might regulate and synchronize the activity in odor triggered third order neurons, similar to the function suggested for other GABAergic neurons (Honeybee: Grünewald, 1999; Ganeshina and Menzel, 2001; Szyszka et al., 2005. Locusts: Perez-Orive et al., 2004).

### The protocerebral neurons

The two MB extrinsic neurons receiving information in the medial lobe of the MB had axonal projections in the SP and the LP, respectively which partly overlapped with the projections of the ml-APT neurons (Figures 8A–G and 10A–F). A variety of MB extrinsic neurons have previously been described in several insect species like the honeybee, the fruit fly, and the cockroach (Honeybee: Homberg, 1984; Mauelshagen, 1993; Rybak and Menzel, 1998; Strausfeld, 2002. Fruit fly: Ito et al., 1998; Tanaka et al., 2008. Cockroach: Li and Strausfeld, 1997). Although we did not find a complete morphological resemblance, the two stained extrinsic neurons in this study (Figures 8A–G) made connections between the lobes of the MB and the LP and SP, like neurons described in the honeybee (Strausfeld, 2002) and in the fruit fly (Ito et al., 1998). For instance, efferent MB extrinsic neurons in the fruit fly connect mainly the head of the α lobe with the SP and the LP (Ito et al., 1998), whereas the stained MB extrinsic neurons in this study projected exclusively from the swellings of the medial lobes (β and β′) to the SP and the LP. Dendritic arborizations confined to the swellings of the lobes seemed to be in line with findings in the fruit fly (Ito et al., 1998).

Mushroom body extrinsic neurons, including the PE1 neuron in the honeybee, have been shown as multi modal as well as responding to a variety of odor stimuli (Homberg, 1984; Mauelshagen, 1993; Rybak and Menzel, 1998; Li and Strausfeld, 1999; Okada et al., 2007). Our recordings in *H. virescens* showed that only one of the two MB extrinsic neurons responded to the mechanical stimulation (air puff, Figures 6B,C) but not to stimulation with tastants. However, since stimulation with tastants was only tested for the MB-LP neuron, we cannot conclude whether other medial lobe extrinsic neurons respond to this modality. In respect to odor stimuli both MB extrinsic neurons responded to all applied odorants, but with different response profiles [neuron N5 (MB-SP) and N21 (MB-LP) in Figures 3A,B and 6B,C, respectively]. A clear difference in response strength to stimulation with the single odorants and the blends appeared in the MB-SP neuron (Figure 6B). This implies an odor specific response to the primary odorants when presented in a novel non-associative situation. More recordings are obviously necessary to generalize this as a principle for the medial lobe extrinsic neurons in *H. virescens*. However, in other insect species odor specific responses among cells of the MB, including extrinsic neurons has previously been reported (Honeybee: Szyszka et al., 2005. Locust: MacLeod and Laurent, 1996; Perez-Orive et al., 2002; Stopfer et al., 2003; Cassenaer and Laurent, 2007. Fruit fly: Turner et al., 2008). Since MB extrinsic neurons probably are involved in mediating associative olfactory information, it would be interesting in future experiments to test MB extrinsic neuron responses to the primary odorants before and after conditioning in *H. virescens*.

Another interesting question is whether the olfactory specificity or the odor code is preserved through the neural network of the olfactory system from the receptor neuron input to specific glomeruli in the AL and further to the descending neurons in the pre-motor area ventrally of the LP. In this study we have shown that the information about a plant odor blend is being processed in parallel through multiple pathways. Thus the SP receives information about the complex PB10 blend both directly from the AL and indirectly from the MB and the LP, whereas the LP receives the same information directly from the AL via all three APTs and indirectly from the MB (Figures 10A–F). Whether other parallel routes exist in *H. virescens* is not known. However, in other insects additional pathways to the MB have been described (Ganeshina and Menzel, 2001; Perez-Orive et al., 2002; Keene and Waddell, 2007). One implication of parallel olfactory pathways might be to process information in a context dependent manner in the different areas. Based on many studies it is previously suggested that whereas the MB function as a coincidence detector for associative olfactory memories, the AL-LP pathway represent a novel or an inexperience pathway for olfactory dependent behavior (Heimbeck et al., 2001; Tanaka et al., 2004; Keene and Waddell, 2007). In the fruit fly it is speculated whether the LP/LH is also involved in olfactory associative memories (Wang et al., 2003b).

In this study the mass and the single cell staining indicates a possible overlap of the most anterior-ventrally projections of PNs in the l- and the ml-APT and the dendritic arborization of the LP-descending neuron (Figures 11A–C). This suggests that the output area of the LP is located anterior-ventrally of the OA. Axonal projections of a taste responding neuron have previously been shown to project in this area implying a possible integration of the two chemosensory modalities (Kvello et al., 2009). Integration of multimodal information in the output areas of LP has also been suggested in flies, indicated by visual and olfactory projections to this area (Strausfeld, 1976; Tanaka et al., 2004). Unfortunately, the descending neuron in this study was only tested for olfactory (inhibitory) and mechanical (no response) input and not with gustatory or other modalities. The strong inhibitory responses particularly to the blend (Figure 6E) might be mediated directly from PNs in the ml-APT, from third order LP neurons as described in the fruit fly (Tanaka et al., 2004; Jefferis et al., 2007), or from the MB-LP route. Although the axonal projections of the presented MB-LP neuron in this study did not overlap with the dendritic arborizations of the descending neuron, other MB-LP neurons might overlap. The inhibitory response of the descending neuron is interesting in connection with the speculation by Okada et al. (2007) that inhibition in these neurons might be mediated directly or indirectly from MB extrinsic neurons in naïve individuals and reduced following olfactory learning. The challenge of further studies would be to test responses to primary odorants and mixtures before and after learning to find out whether and how the inhibitory responses changes in descending neurons. The result that only mixtures, especially the PB10, elicited strong inhibitory responses in the descending neuron is interesting in relation to behavioral results in the hawk moth. Here complex mixtures derived from host plants and not the single constituents elicit feeding behavior (Riffell et al., 2009a,b). This implies that odor responses in descending neurons are elicited upon co-activation of multiple inputs from parallel pathways in the direct AL-LP stream (inexperienced) and/or from the integrative experienced AL-MB-LP stream.

## Conflict of Interest Statement

The authors declare that the research was conducted in the absence of any commercial or financial relationships that could be construed as a potential conflict of interest.

## Author Note

High resolution figures, reconstructions, and files from the standard brain project are available by contact at the following blog address: http://olfactoryblog.wordpress.com/or by email: olfactoryblog@gmail.com
